# Coaching as a Model for Facilitating the Performance, Learning, and Development of Palliative Care Nurses

**DOI:** 10.1177/23779608221113864

**Published:** 2022-07-15

**Authors:** Cristina Costeira, Maria A. Dixe, Ana Querido, Joel Vitorino, Carlos Laranjeira

**Affiliations:** 1 386407 School of Health Sciences of Polytechnic of Leiria, Leiria, Portugal; 2 467343 Centre for Innovative Care and Health Technology (ciTechCare), Leiria, Portugal; 3 451281 The Health Sciences Research Unit: Nursing (UICISA: E), Nursing School of Coimbra (ESEnfC), Coimbra, Portugal; 4 451731 Center for Health Technology and Services Research (CINTESIS), NursID, University of Porto, Porto, Portugal; 5 Palliative Care Service of Portuguese Oncology Institute of Coimbra, Coimbra, Portugal; 6 451287 Research in Education and Community Intervention (RECI I&D), Piaget Institute, Viseu, Portugal

**Keywords:** coaching, mental health, palliative care, nurse leaders, evidence-based practice

## Abstract

Palliative care nurses experience huge pressures, which only increased with coronavirus disease 2019 (COVID-19). A reflection on the new demands for nursing care should include an evaluation of which evidence-based practices should be implemented in clinical settings. This paper discusses the impacts and challenges of incorporating coaching strategies into palliative care nursing. Evidence suggests that coaching strategies can foster emotional self-management and self-adjustment to daily life among nurses. The current challenge is incorporating this expanded knowledge into nurses’ coping strategies. Coaching strategies can contribute to nurses’ well-being, empower them, and consequently bring clinical benefits to patients, through humanized care focused on the particularities of end-of-life patients and their families.

## Introduction

Palliative care (PC) aims to enhance the quality of life of patients facing life-threatening diseases, as well as improve the well-being of their carers and significant others, through active holistic care (Radbruch et al., 2020). This imposes serious challenges for nurses, such as end-of-life (EOL) decisions and contact with suffering and dying people (Parola et al., 2018). Working in a stressful environment can lead to high levels of emotional exhaustion, job insecurity, and decreased quality of care when not effectively managed (Gómez-Urquiza et al., 2020; McKinless, 2020). Alarming statistics about health care provider burnout indicate a growing need for an emphasis on self-care and recognizing that professionals must attend to their own well-being (Leo et al., 2021; Tawfik et al., 2019).

Workplace well-being is a key issue in the field of health care (Ali et al., 2021). Thus, nursing managers should mobilize strategies that promote well-being and help their nurses develop resilience and facilitating skills to prevent burnout, turnover, job dissatisfaction, and mental illness (Shah et al., 2021; Trepanier et al., 2022). The COVID-19 pandemic has brought increased constraints, caused by a personal life unbalanced by professional demands (Chidiebere Okechukwu et al., 2020; Shah et al., 2021).

Coaching is evolving as a professional skill and has been recommended for nurses exposed to high stressors, particularly in order to empower and guide nurse leaders in supporting their respective nurses (Piamjariyakul et al., 2019; Trepanier et al., 2022). Coaching can influence the health care environment within the field of PC, implying that professionals must understand themselves and behave as part of a multidisciplinary team (Suikkala et al., 2021).

This paper discusses the role of coaching in PC nursing, presents the guiding principles of coaching, and analyzes some applications in palliative nursing practice, education, and leadership. Finally, trends in coaching for EOL and PC are systematized.

## Brief Review

**Figure 1.**
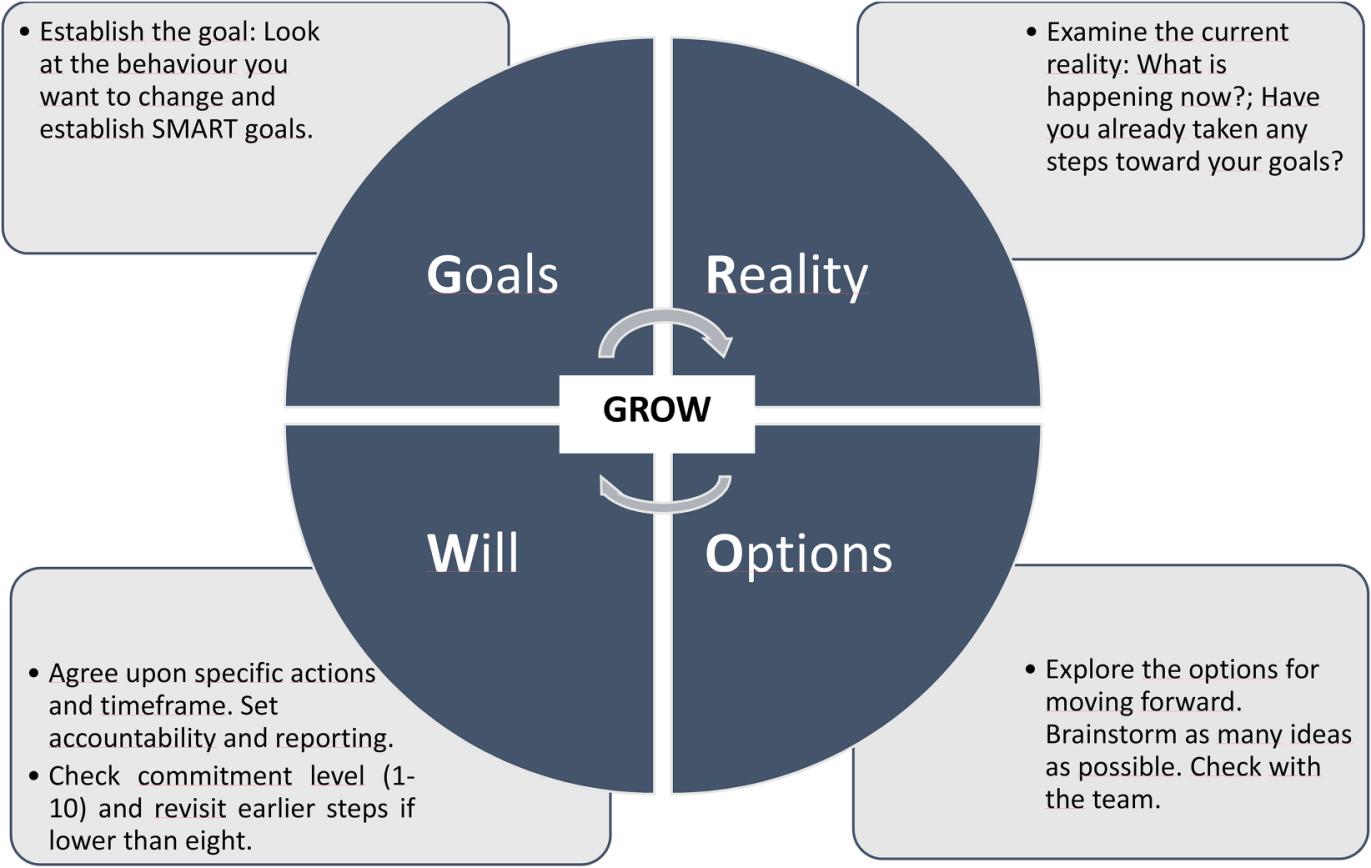
Grow model according to Whitmore (2017).

The concept of coaching is often confused with mentoring, but they are two different concepts. Coaching is defined as an interactive and interpersonal process that offers personal and professional support through the acquisition of knowledge, skills, and fundamental actions for professional practice (Sarroeira et al., 2020). The coaching process promotes self-knowledge and the establishment of goals through a deep analysis of the individual, provoking a reflection on perspectives, mindsets, beliefs, and approaches that can lead to more sustainable behavior (Cable & Graham, 2018; Whitmore, 2017). Typically, coaching involves one-to-one learning during a short period. Widely used to structure a coaching conversation, the Goal, Reality, Options, Will (GROW) model (Figure 1) is a succinct framework for coaching grounded on learning through experience: reflection, insight, making choices and pursuing goals. According to Rodenbach et al. (2020), coaching PC conversations could increase health care providers’ preparedness in discussing PC topics and completing goals of care. In contrast, mentoring implies a long period during which the mentor shares their experience and knowledge to help the individual in their development process (Kowalski, 2019; Seehusen et al., 2021). Mentoring is typically less structured than coaching, the latter following a more non-directive and rigorous structure. In mentoring, while having a mentoring agenda of meetings and goals is recommended, the mentee is responsible for this organization.

### Applications of Coaching in Palliative Nursing Practice, Education, and Leadership

The concept of coaching in nursing was introduced by Benner (1984). She used the Dreyfus Model to explain how nurses progressively develop skills, which included coaching as a strategy (Potter, 2019). More recently, the Theory of Integrative Nursing Coaching was developed to guide coaching practice in nursing, education, research, and health policy (Moore et al., 2022). This middle-range theory focuses essentially on the relationship with the person and emphasizes self-care practices, well-being, intentionality, presence, attention, and therapeutic use of oneself as fundamental means to facilitate recovery. Nurse coaches, by facilitating personal transformation through listening with Healing, Energy, Awareness, Resiliency, Transformation (HEART), progress toward the aim of nourishing people in a sustainable world—from the microcosm to the macrocosm (Moore et al., 2022).

Coaching can be applied to nurses (Dyess et al., 2017; Yusuf et al., 2018), nurse leaders (Waldrop & Derouin, 2019), nurse students (Hurley et al., 2020; Kaldawi, 2022; Sezer & Şahin, 2021) and nursing care support with patients and their families (Flanagan et al., 2022; Johansson et al., 2020; Singh et al., 2022), the latter being the most frequent application. Coaching assists nurses in engaging in dialogs and interactions aimed at improving professional growth, career commitment, and practice (Barr & Tsai, 2021). Thus, Donner and Wheeler (2009) propose a classification of coaching in four domains, namely:

1. *Peer coaching*. Can be applied to assist nurses in furthering their careers and increasing job satisfaction. This area is one of the least applied. Peer coaching has been successfully used in inpatient settings to teach primary PC skills (Jacobsen et al., 2017).
2. *Health coaching*. A valuable method for nurses who want to assist patients in reaching their aspirations. This focus area is very important to PC nurses who are challenged daily to help patients and families accomplish their last wishes. Coaching conversations should include several communication skills (such as therapeutic presence, deep listening, use of silence and motivational interviewing) in order to help patients identify their individual barriers and facilitators and realize their goals (Rosa et al., 2021a). Coaching can help nurses adopt a person-centered practice but can also be used to prevent ill health and reduce the impact of chronic conditions (Barr & Tsai, 2021; Benzo et al., 2017; Fazio et al., 2019; Singh et al., 2022).
3. *Interprofessional coaching*. The emphasis is on advancing interprofessional education and practice, promoting teamwork, and ensuring the team provides comprehensive care. This area fosters the development of professional skills such as multi-professional communication (Ahmann et al., 2021; Douglas & MacPherson, 2021; McKinless, 2020; Niglio de Figueiredo et al., 2015) and is a promising area in PC.
4. *Succession planning*. Coaching can be used to help plan successions, facing the changing definitions of work-life balance, the demographic reality and the impending retirement of significant numbers of leaders. Planning for leadership succession is becoming a key component of long-term human resources strategies in many organizations. Since leadership plays an essential role, affecting patients and professional outcomes and even the work environment (Specchia et al., 2021), nurse leaders must develop facilitators skills, especially for less experienced nurses and those beginning their careers (Major, 2019). Leaders have a particularly influential role in the implementation of evidence-based practices by providing a supportive culture and environment (Bianchi et al., 2018).

Other models, such as positive psychological coaching (PPC), have emerged as a new paradigm for practitioners interested in professional development (Richter et al., 2021). Also known as strengths-based coaching, PPC “can be defined as a short to medium-term professional, collaborative relationship between a client and coach, aimed at the identification, utilization, optimization, and development of personal strengths and resources in order to enhance positive states, traits and behaviours” (van Zyl et al., 2020, p. 1). A recent study produced a 5-phase PPC model aimed at promoting professional development (Richter et al., 2021).

Both coaching in nursing and the positive psychology model recommend best practices that organizations should incorporate to develop professional staff resiliency, leadership effectiveness, and enhanced team performance (Dyess et al., 2017; Grant & Atad, 2021; Spiva et al., 2021). In this sense, some components, tools, and evaluation strategies of coaching training programs (Table 1) should be used to improve collaborative teamwork (Johansson et al., 2020; Penconek et al., 2021; Yusuf et al., 2018), strengthen team relationships, and promote engagement with the institution (Dyess et al., 2017; Johnson et al., 2020). The current challenge is incorporating this expanded knowledge into nurses’ coping strategies. The nursing profession must make strategic plans that develop skills in nursing education, clinical practice, health policy, and even research. Nurses in PC are well-positioned to develop and implement coaching tools as a means to improve their mental health and professional soft skills needed for success on the job (such as empathy, self-motivation, resilience, emotional literacy, critical thinking and work ethic) (Table 2).

**Table 1.** Comparison of Key Components and Coaching Strategies of Different Training Programs.

	Coaching training program for nursing (Donner & Wheeler, 2009)	Positive psychological coaching (Richter et al., 2021; van Zyl et al., 2020)
Content/coaching phases	(1) Planning (2) Implementing (3) Evaluating (4) Sustaining • Coaching functions and competencies • Coaching process • Managing typical coaching scenarios	(1) Creating the relationship (2) Strength profiling and feedback (3) Developing an ideal vision (4) Realistic goal setting, strategizing, and execution centered around strengths (5) Concluding or re-contracting • Continuous process 1: Learning transfer • Continuous process 2: Action tracking and continuous evaluation • Continuous process 3: Empowerment (reframing, reinforcement)
Learning strategies (techniques and tools)	Supporting documents to complement in-class instruction Practice, observation and feedback sessions from a coaching expert Ongoing access to and consultation with an expert coach while skills are developed in practice settings (face-to-face meetings, teleconferences, e-mail) Periodic review sessions to facilitate reflection on learning and/or the coaching relationship Mid- and/or end-of-program seminars to continue the development of coaching skills, share success stories/strategies	• Interpersonal relation [personalized contracting] • Socratic goal setting • Strengths spotting • Self-compassion • Active listening • Using appreciative inquiry (4D-cycle) • Personal resource mapping • Guided self-reflections • Reframing negative narratives • Self-administered intentional activities: hope exercises, gratitude • Homework between sessions to increase adherence and engagement. • Creating a personal development plan
Evaluation strategies	Satisfaction surveys (coach and client feedback) Coaching performance evaluations (pre-/post-training outcomes) Indicators of organizational impact (e.g., data retention, staff satisfaction)	• Hope assessment tools • Strengths-focused psychometric assessments • Assessment of the learning transfer

**Table 2.** Skills Improved by Coaching Tools.

Observation skills, providing feedback, questioning, leadership, communication, trust-building, problem definition and problem-solving, decision making and conflict management (Donner & Wheeler, 2009) Improve relationships with clinicians (Yusuf et al., 2018) Develop communication skills (Douglas & MacPherson, 2021) Promote best clinical practice in clinical contexts by patients (Johansson et al., 2020) Facilitate engagement and peer learning (Johnson et al., 2020) Promote a positive workplace culture (Dyess et al., 2017) Reinforce emotional intelligence skills (McKinless, 2020) Mitigate burnout, hold more rewarding, person-centered conversations in clinical practice and enable flexible responses and constructive adaptation to change (Maini et al., 2020) Improve social support, team cohesion, and wellbeing (Penconek et al., 2021) Support goal attainment, self-insight, psychological well-being, and solution-focused thinking; enhance personal agency through goal-focused self-regulation (Grant & Atad, 2021) Prepare for entering the job market (Hurley et al., 2020)

## Current Insights and Trends in Coaching for EOL and PC

Clinical environments affect the safety and mental health of nurses as well as the quality of their health care service. The quality of this environment should not be neglected by health decision-makers. Coaching as a process of nurse development and empowerment is a powerful ally (Dossey & Luck, 2015). Used frequently in health care delivery, only rarely is it used to promote the well-being of health professionals. Health care organizations are still very focused on the needs of patients and improving care outcomes while neglecting the needs of health professionals. This may lead to medium and long-term complications in nurses’ health and well-being, and consequently, the care they provide (Rosa et al., 2021a).

The significant advantages of coaching for nurses, especially those subject to high work stressors (i.e., PC nurses), can be obtained using simple and accessible tools during different phases of nurse education. This demands partnership and collaboration between nursing schools and health institutions to improve professional performance, as well as nurse well-being and mental health.

With the COVID-19 pandemic, virtual and mobile phone-based coaching interventions have become more common (Rosa et al., 2021b; Tsiouris et al., 2020; Wittenberg et al., 2018). The added value of such approaches in terms of effectiveness, technology acceptance, and reduced costs has been well documented (Tsiouris et al., 2020). Virtual coaching “enhances personal well-being, improves global health partnerships and knowledge exchange, and fosters communication across all levels of education and clinical practice” (Rosa et al., 2021b, p. 1).

## Conclusion

The coaching model offers a systematic, relational strategy that includes tools to enhance nurse competencies, both individual and team skills such as self-analysis, therapeutic presence, compassion, and moral insight for the attainment of specified PC goals. The emphasis is on individual needs, strengths, and shortcomings, employing dialog and reflection in a setting of confidentiality and trust. This method is very beneficial for one-on-one “skill-based” teaching and learning. It also helps the PC team handle communication and psychological concerns while offering emotional support to professionals. Leaders play an, especially, essential role, as they are the most familiar with their employees and can thus best adjust and adopt those tools.
